# Guidelines on "Standards of management of idiopathic scoliosis with corrective braces in everyday clinics and in clinical research": SOSORT Consensus 2008

**DOI:** 10.1186/1748-7161-4-2

**Published:** 2009-01-16

**Authors:** Stefano Negrini, Theodoros B Grivas, Tomasz Kotwicki, Manuel Rigo, Fabio Zaina, the international Society on Scoliosis Orthopaedic and Rehabilitation Treatment (SOSORT)

**Affiliations:** 1 ISICO (Italian Scientific Spine Institute), Via Bellarmino 13/1, 20122 Milan, Italy; 2 Orthopaedic Department, "Thriasio" General Hospital, G. Gennimata Av. 19600, Magoula, Attica, Greece; 3 Department of Paediatric Orthopaedics, University of Medical Sciences, Poznan, Poland; 4 Istituto Èlena Salvà, Barcelona, Spain

## Abstract

**Background:**

Reported failure rates,(defined based on percentage of cases progressing to surgery) of corrective bracing for idiopathic scoliosis are highly variable. This may be due to the quality of the brace itself, but also of the patient care during treatment. The latter is sometimes neglected, even though it is considered a main determinant of good results among conservative experts of SOSORT. The aim of this paper was to develop and verify the Consensus on management of scoliosis patients treated with braces

**Methods:**

We followed a Delphi process in four steps, distributing and gradually changing according to the results a set of recommendations: we involved the SOSORT Board twice, then all SOSORT members twice, with a Pre-Meeting Questionnaire (PMQ), and during a Consensus Session at the SOSORT Athens Meeting with a Meeting Questionnaire (MQ). We set a 90% agreement as the minimum to be reached.

**Results:**

We had a 71% response rate to PMQ, and 66.7% to MQ. Since the PMQ we had a good agreement (no answers below 72% – 70.2% over 90%). With the MQ the agreement consistently increased for all the answers previously below 90% (no answers below 83%, 75% over 90%). With increasing experience in bracing all numerical criteria tended to become more strict. We finally produced a set of 14 recommendations, grouped in 6 Domains (Experience/competence, Behaviours, Prescription, Construction, Brace Check, Follow-up).

**Conclusion:**

The Consensus permits establishment of recommendations concerning the standards of management of idiopathic scoliosis with bracing, with the aim to increase efficacy and compliance to treatment. The SOSORT recommends to professionals engaged in patient care to follow the guidelines of this Consensus in their clinical practice. The SOSORT criteria should also be followed in clinical research studies to achieve a minimum quality of care. If the aim is to verify the efficacy of bracing these criteria should be companions of the methodological research criteria for bracing proposed by other societies.

## Background

**Figure 1:**
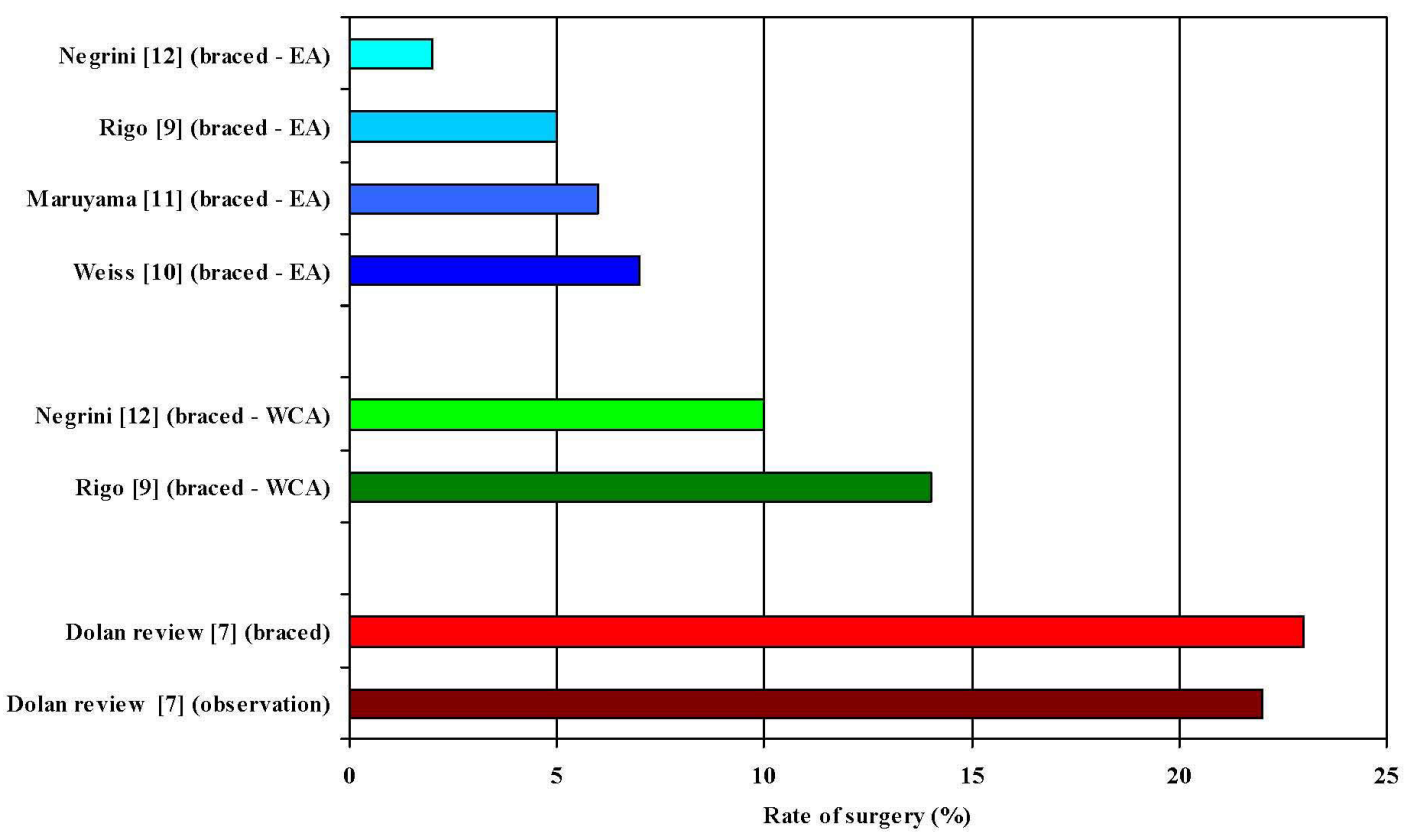
**Rates of surgery in scoliosis over 30°**. Rates of surgery, with and without bracing (without exercises), in scoliosis over 30° reported in a metanalysis by Dolan and Weinstein [7], compared with results of bracing plus exercises in scoliosis over 30° published by SOSORT members [8-12] in terms of Efficacy Analysis (EA) and Worst Case analysis (WCA).

Bracing today can be considered a worthwhile treatment for adolescent idiopathic scoliosis (AIS): the strength of evidence [1] of this recommendation is grade B [2,3]. The existing Guidelines support their use [4,5]. Nevertheless, doubts have been raised from a series of authors [6,7]. Recently, a metanalysis of the English literature on bracing has been published [7]. Outcomes for bracing, or observation only, were compared. The authors concluded that "Based on the evidence presented here, one cannot recommend one approach over the other to prevent the need for surgery in AIS." Nevertheless, the authors elected to exclude from the study the groups with bracing plus exercises. Under the conditions of their analysis, therefore, according to the Material and Methods of the paper, their conclusion should have been as follows: "Based on the evidence presented here, *according to the English literature and excluding the combined approach of bracing and exercises*, one cannot recommend one approach over the other to prevent the need for surgery in AIS.". In fact, according to the same criteria used in the previously mentioned metanalysis [7], the papers published by some members of the international Scientific Society on Scoliosis Orthopaedic and Rehabilitation Treatment (SOSORT) [8-12], that are in the English literature but include also exercises, have yielded results that are in conflict with those of the reported systematic review [7] (Figure 1).

The world of treatment of scoliosis is gradually changing [2] and two main ideas are facing each other: one is more surgically oriented, with the prevalent idea that bracing is not an effective treatment [6]. This position has been used to justify the ethical approval of a Randomised Clinical Trial now underway in the US. The SOSORT is more conservatively oriented, and their members have presented a substantial body of data on the effectiveness of conservative treatment in general [8-12], and of exercises and bracing in particular [13-26]. Consequently, a formal debate among this Society concluded that a Randomised Controlled Study on brace efficacy would be ethically unacceptable [27]. During the Boston SOSORT Meeting this distinct ethical approach among different Societies was discussed. A critical need to define the criteria for success of brace treatment of scoliosis was identified, [27]. In fact, there was the general acknowledgement that the differences in the literature are on technical as well as management factors. In other words, bad results of bracing can be due to bad braces (and this could be verified through in-brace x-rays to check the correction obtained), or to improper management of the patient. This factor can ultimately influence compliance. The latter has not been yet sufficiently stressed in the literature despite its critical role in efficacy of any treatment [3,28,29].

Recently, methodological criteria for brace studies have been defined [30]. These criteria are key scientific points, and should be followed when proposing final results of treatment whenever possible even if research in this field is so scanty that other preliminary results are still needed to refine the approach. On the other hand there is the lack of methodological criteria of the treatment itself, that is today based on many different braces [3], without a good international codification [31], and without clearly defined and common approaches for mechanical action [27]. In fact, despite these technical differences, it was quite clear that among conservative experts of SOSORT there were strong similarities in management of patients that could greatly contribute to success of treatment, as we can see everyday in our clinical practice, and previously published [8-12].

The aim of this work was to define standards of management of AIS with corrective braces to be applied in everyday clinics but also in clinical research. In this paper we will present the results of this process and the final recommendations to the entire scientific scoliosis community coming from the conservative experts of the SOSORT.

## Methods

We followed a Delphi process as described by Jones and Hunter [32]. The first draft of recommendations (Additional file 1) has been circulated in the SOSORT Board (Table 1) to be changed/integrated. The aim was to have up to 10–15 recommendations grouped together under a limited number of headings and statements. The final set of recommendations has been circulated to the SOSORT Board to be finally approved. We then sent out a questionnaire to all SOSORT members (Pre-Meeting Questionnaire – PMQ) (Additional File 2), including 14 recommendations grouped in 6 Domains (Experience/competence, Behaviours, Prescription, Construction, Brace Check, Follow-up). Each member ranked each recommendation and statement in the questionnaire according to some ordinal scales, explained their ranking, proposed changes or deletions of each statement in the recommendation, and added any comment they wanted.

**Table 1:** Board of SOSORT that prepared and approved the first draft of the questionnaire.

**Board of SOSORT**	
Manuel Rigo	President
Theodoros B. Grivas	Next President
Tomasz Kotwicki	Past President
Stefano Negrini	Secretary
Elias Vasiliadis	Treasurer
Hans Rudolph Weiss	
Toru Maruyama	
Joe P. O'Brien	
Tamar Neuhaus	

During the Athens Meeting of SOSORT a full presentation of the results obtained during this process has been made. All the answers that did not reach at least a 90% agreement during the previous rounds where discussed and voted in a final questionnaire (Meeting Questionnaire – MQ) in a session reserved only to SOSORT Members (Additional file 3).

We divided the responders according to profession and number of braces prescribed per year and analysed the differences among these sub-groups. We applied parametric and non-parametric tests according to what required and set statistical significance at α = 0.05.

## Results

Among SOSORT Members we had a 71% response rate to PMQ, and 66.7% to MQ (Table 2), with no differences in any of the baseline values (Table 3).

**Table 2:** SOSORT Members who responded to the Pre Meeting, and to the Meeting Questionnaire and accepted to be cited.

		**Pre-Meeting**	**Meeting**
Atanasio	Salvatore	X	
Auler		X	
Aulisa	Lorenzo	X	
Betts	Tony	X	
Bowman	Gez	X	
Dallmayer	Robert		X
De Mauroy	Jean Claude	X	X
Durmala	Jacek	X	X
Egarter	Vurt		X
Ferraro	Claudio	X	
Gallo	Dino		X
Gil	Jose	X	
Grivas	Theodoros	X	X
Herling	Orna		X
Iemolo	Biagio	X	
Janssen	Beth	X	
Kotcwicki	Tomas	X	X
Landauer	Franz	X	X
Lior-Neuaus	Salum	X	X
Lou	Edmund	X	X
Mann	Kevin		X
Marianthi	Tzara		X
Marti	Cindy	X	
Maruyama	Toru	X	X
Meller-Gattenyo	Liat		X
Menko	Yolanda		X
Monica	Magras		X
Negrini	Stefano	X	X
Negrini	Alessandra	X	
Neuhaus	Tamar	X	X
O'Brien	Joseph	X	X
Parzini	Silvana	X	
Plumis	Avraam	X	
Quera Salva	Gloria		X
Rainero	Giovanni	X	
Rigo	Manuel	X	X
Romano	Michele	X	X
Rualm	Chales H.		X
Sancho	Joaquin		X
Sepin	Winfried		X
Sooba	E		X
Stikeleather	Luke	X	X
Tedeschi	Claudio	X	
Tessadri	Fabrizio	X	
Tielen	Eric		X
Vasiliadis	Elias	X	X
Weiss	Hans Rudolf	X	X
Wynne	James H.	X	X
Zaina	Fabio	X	X

**Table 3:** Characteristics of the population who answered to the Pre-Meeting and the Meeting Questionnaires.

		**Pre Meeting**	**Meeting**	**Test**
**Gender**	*Males*	27	24	NS
	*Females*	7	9	

**Age**	*(av +/- sd)*	46.6 +/- 8.6	44.5 +/- 9.3	NS

**Experience**	*(av +/- sd)*	18.3 +/- 10.6	18.3 +/- 10.3	NS

**Braces/y**	*(med – 95% IC)*	150 (30–965)	200 (28–1725)	NS
	*Less than 100*	8	5	
	100–300	15	12	
	*Over 300*	5	9	

**Profession**	*MD-OS*	12	9	NS
	*MD-PRM*	6	9	
	*CPO*	5	8	
	*PT*	9	7	
	*Oth*	2	2	

**av **average; **sd **standard deviation; **med **median; **95% CI **95% Confidence Interval; **MD-OS **Physician, Orthopaedic Surgeon; **MD-PRM **Physician, Rehabilitation Specialist; **CPO **Orhotist; **PT **Physiotherapist; **Oth **Others

Overall the answer to the PMQ revealed a good agreement among SOSORT members, with no answers below 72%, and 70.2% of answers over 90% of agreement (Table 4). Most of the disagreements related to the first two (Domain Experience/competence) and the last two (Domain Follow-up) recommendations. In general, only one item (#13) was considered by at least 10% of responders as "could be recommended" or less, while all other items were considered "recommended" or "highly recommended." Most of the problems related to the "personal behaviour" answers, that were not always correspondent to the recommendations, and the possible usefulness for "research application." During this stage a lot of comments were received (Table 5), and recommendations were changed accordingly to be proposed in the MQ.

**Table 4:** Answers to the Pre-Meeting Questionnaire revealed a good agreement among SOSORT members, with no answers below 72%, and 70.2% of answers over 90% of agreement.

**Domain**	**Rec**	**Agr**	**Imp**	**Type of rec**	**Per**	**Clin**	**Res**	**Details (at least 90% agreement)**	
**Experience**	1	85%	85%	94%	75%	90%	80%	5 years to be a master	2 years with a master
**Competence**								3 years of practice	
								1 brace per week	last 2 years
								2 evaluations per week	last 4 years
	
	2	85%	85%	90%	84%	90%	83%	2 years with a master	
								2 years of practice	
								2 braces per week	last 2 years

**Behaviours**	3	97%	94%	91%	88%	87%	83%		
	
	4	97%	97%	94%	93%	93%	79%		
	
	5	97%	97%	91%	94%	90%	86%	MD prescription	
								CPO construction	
								MD check with CPO	

**Prescription**	6	91%	97%	94%	87%	94%	86%	Details of construction	
								Convinced & committed	
								Compliance	

**Construction**	7	97%	97%	94%	94%	94%	94%	Check & discuss	
								Fully execute	

**Brace check**	8	100%	97%	94%	94%	97%	93%	Individual needs	
								3D correction	
								Aesthetic correction	
								Tolerability	
								Apply changes	
								Counselling	
	
	9	97%	94%	97%	94%	94%	90%		

**Follow-up**	10	100%	97%	97%	94%	97%	90%		
	
	11	97%	97%	97%	97%	97%	90%		
	
	12	97%	97%	94%	97%	97%	90%		
	
	13	79%	79%	87%	74%	80%	72%		
	
	14	81%	81%	94%	81%	90%	83%		

**Rec **Recommendation; **Agr **Agreement; **Imp **Importance; **Per **Personal behaviour; **Clin **Clinical application; **Res **Research application

**Table 5:** Comments received to the Recommendations of the Pre-Meeting Questionnaire.

**Domain**	**Recommendation**	**Comments**
**Experience**	1	Not all centres have this many patients but may still provide a local service? it is difficult to be so dogmatic with numbers
**Competence**		The number of treatments required is too high for a small country
		Many little centres are not able to fulfill these criteria
		Quantity is not always reflecting quality
		Experience is important but it is not the only thing behind correct clinical decisions
		This is an ideal, but if there is a good CPO and the MD do not fulfill this recommendation is this important?
		My concern is availability of master physicians (there aren't in some countries)
		You cannot prohibit prescriptions by graduated MDs
	
	2	Experience is important but it is not the only thing behind correct clinical decisions
		This can be a problem for little centres or to recruit new CPOs
		My concern is availability of master physicians (there aren't in some countries)
		You cannot prohibit construction by licensed CPOs
		Team work for private professionals can be a problem

**Behaviours**	3	Working as a team is difficult when you are not in the same place
		The team must include PTs specialized in the field
		Ideally the physician, orthotist and therapist should be seeing patients together in a clinic setting but outside of the clinic/hospital based programs this occurs very rarely
		Although ideal, I believe this will seriously impede the development of scoliosis interest in US by independent individual professionals (CPO and PT). This will run the risk of scoliosis only being able to be treated by bigger institutions and "directed" by MD. It could really impede the motivation of new and very strong independents from getting involved with scoliosis and with SOSORT. I believe you can have for instance a MD who is supportive of conservative management and refers to a very good CPO and appropriately defers the brace making and corrections to the very skilled CPO.
		Quite frankly, although the MD is important in diagnosis and especially in medical differential diagnosis, once he/she is done with that job, MUCH of the remaining hands-on conservative management is the responsibility of the CPO (and PT). These people should be allowed to have some degree of independence. In order to attract and retain really great CPOs, and PTs, these people will want to over time be more than just "technicians" They will want to have a decision making and larger role in the overall management of the case... Of course along side the MD is best, but not under the order of the MD...
	4	To increase compliance MDs shouldn't change during treatment
		Sometimes psychologists can be useful
		Also PTs have an important role in this
		Sometimes CPOs can create problems if they do not satisfy previous recommendations
		Compliance is a problem when you receive negative messages inside the team
	
	5	If CPO knows his work is the MD that has to discuss with him
		CPOs should lead this action and not MDs
		In North America ortho surgeons do not write details of prescription
		US physicians are not trained in giving details of brace prescription and this should be done together by CPO and MD
		If an MD is not convinced and committed he should send the patient to another MD for treatment
		In some brace prescriptions you don't need to give details
		Any action to promote compliance should be not manipulative (e.g. "if you do not wear a brace you will finish in a chair"...)
		Don't mix compliance with prescription

**Prescription**	6	If CPO knows his work is the MD that has to discuss with him
		CPOs should lead this action and not MDs
		In North America ortho surgeons do not write details of prescription
		US physicians are not trained in giving details of brace prescription and this should be done together by CPO and MD
		If an MD is not convinced and committed he should send the patient to another MD for treatment
		In some brace prescriptions you don't need to give details
		Any action to promote compliance should be not manipulative (e.g. "if you do not wear a brace you will finish in a chair"...)
		Don't mix compliance with prescription

**Construction**	7	In US details are not given by the MDs
		Don't mix compliance with construction
		About compliance CPOs have not to act autonomously but eventually support the action of MDs
		Any action to promote compliance should be not manipulative (e.g. "if you do not wear a brace you will finish in a chair"...)

**Brace check**	8	MD has to check and the CPO only for his competence
		This is lead by CPO and MD is not necessary
		It's possible that the check by MD is not immediate
		The other members of the team should give their comments (PTs)
		What does "check aesthetic correction" mean?
	
	9	We do not check braces but patients in brace
		Clinical variance (no consensus)
		1.I check only clinically, rarely radiographically
		2.I check only radiographically
		3.I check radiographically only the first brace
		4.I check always radiographically a new brace
		The eventual radiographic check should be postponed one month after brace wearing
		We need more details on eventual x-ray check to have a standard
		Who evaluate the brace?

**Follow-up**	10	If PT treatment is twice a week, it is a waste of time to check every time
		PTs usually are not well prepared: this recommendation is possible only in Scoliosis Centres
		This is not research, is only clinics
	
	11	Follow-up must be maximum in 3 months, in growth spurt in 2 months (push loosening)
		Controls in 3 months in case of: 1. first brace; 2. growth spurt; 3. progressive curve; 4. atypical curve; 5. predicted poor compliance; 6. request of other team members (CPO, PT)
		It's important to give protocols or use recalls
	
	12	MDs have rarely enough time and knowledge: this should be responsibility of CPOs, that can judge it better
		This must be respected by Health National Services
		Some braces allow more adjustability and should be chosen
		Sometimes in specific braces less than 3 months is necessary
		Correction is another reason to change a brace
	
	13	Control should be made by MDs (CPOs should intervene autonomously only if the brace breaks)
		MD & CPOs should check the braces together
		It's enough that one of the team (MD, PT, CPO) checks the brace regularly (every 2–3 months)
		The problem is growth and not time: I suggest every 2–3 cm. or kg.
		First braces should be checked after 1 month
		Efficacy can be checked only through x-rays and this is not possible every 2–3 months
	
	14	It's not possible because PTs are not inside the hospital team
		PTs usually are not well prepared
		Sometimes PTs tell the patient something wrong !
		If PT treatment is twice a week it is a waste of time
		PTs should tell to physicians and not patients
		This is not research, is only clinics

**General comments**		SOSORT should offer training for MDs, CPOs and PTs
		It's better to compromise for compliance than to be very stiff and loose the patient
		SOSORT should develop methods to detect scoliosis (idiopathic and so on) as a brochure especially for general practitioner and pediatrician
		PTs should be trained in assessment of brace fit
		We need guidelines on x-ray assessment
		We need a group to evaluate brace efficacy and, at the same efficacy, brace compliance
		We need a way to certify MDs, CPOs and PTs prepared in scoliosis treatment
		We need objective questionnaires to evaluate psychological impact of treatments

**MD **Medical Doctor; **CPO **Orhotist; **PT **Physiotherapist

After the previous round and the Consensus and discussion in Athens, the agreement consistently increased (Table 6). Considering that all the questions related only points on which the 90% had not been reached previously, the rate of agreement consistently increased: there were no answers below 83%, and 75% were over 90%. Again, disagreement related particularly to research application, while all recommendations were finally accepted, and in all cases except one, all changes proposed were accepted.

**Table 6:** The agreement among SOSORT members increased in the answers to the Meeting Questionnaire.

**Domain**	**Rec**	**Agr**	**Res**	**General statement**	**Agree on changes proposed**	**Agr on single points**			
						**Point**	**Agr**	**Point**	**Agr**
**Experience**	1	91%	86%	97%					
**Competence**	2	92%	86%	94%					

**Behaviours**	3		91%		97%				
	4		89%		97%				
	5		88%			4	100%	5	94%

**Prescription**	6		86%		91%	1	89%	3	97%

**Construction**	7				97%	1	100%	3	97%

**Brace check**	8				94%	7	100%		
	9				89%				

**Follow-up**	10				94%				
	11				97%				
	12				92%				
	13	94%	83%		91%				
	14	97%	91%		100%				

**Rec **Recommendation; **Agr **Agreement; **Res **Research application

We did not find important differences in the MQ and according to profession in the PMQ In contrast, it was clear that with increasing experience in bracing all the numerical criteria tended to become more strict (Table 7): looking at the group that prescribed/constructed/used less than 100 braces per year versus the over-300 braces per year group, the years required to be a master increased from 1.8 ± 1.0 to 3.4 ± 0.9 respectively. The scoliosis to be evaluated per week doubled from 5.0 ± 2.3 to 9.8 ± 3.0 as well as the braces to be built per week increased from 2.3 ± 1.3 to 5.2 ± 2.7.

**Table 7:** With increasing experience in bracing all the numerical criteria tended to become more strict.

	**Braces per year**	**Less than 100**		**More than 300**		**Statistics**
		*Av*	*Sd*	*Av*	*Sd*	*P*
**Recommendation 1 (physician)**	*Years required to be a master*	1.8	1.0	3.4	0.9	0.023
	*Years of experience with a master*	1.3	0.5	2.6	1.3	0.060
	*Braces prescribed per week*	1.8	1.0	2.0	0.0	0.716
	*Period of brace prescription*	8.1	2.6	10.0	0.0	0.139
	*Scoliosis evaluated per week*	5.0	2.3	9.8	3.0	0.011
	*Period of scoliosis evaluation*	2.0	0.0	2.4	1.8	0.565

**Recommendation 2 (orthotist)**	*Years of experience with a master*	2.9	0.4	3.8	1.1	0.058
	*Years of continous practice in bracing*	1.8	0.5	2.6	1.3	0.273
	*Braces built per week*	2.3	1.3	5.2	2.7	0.029
	*Period of brace building*	2.3	0.8	2.6	1.3	0.693

## Discussion

According to the previous results a final document "Standards of management of idiopathic scoliosis with corrective braces in everyday clinics and in clinical research. The SOSORT Criteria for bracing" was released (Additional file 4).

The SOSORT criteria can serve as

• a clinical guideline for clinicians, including all professionals dealing with scoliosis bracing.

• a tool for patients to check if professionals' behaviours are coherent with the actual gold standard in clinical behaviour.

The SOSORT criteria should be followed in high quality clinical research studies, where the aim is verifying the efficacy of bracing. In this respect, these clinical criteria should be companions of the methodological criteria for bracing published in 2005 [30]. Any future clinical trial (whether randomised or not) should carefully respect these criteria. Otherwise the clinical standards used would not be adequate to assess the efficacy of bracing.

The SOSORT Criteria have been divided into Domains, as follows:

• Experience/Competence (Recommendations 1–2): it is not possible to have any success in bracing without thorough knowledge, coming from a good mastership, and a continuous, thoughtful and dedicated practice;

• Behaviours (Recommendations 3–5): these are central to the success of bracing, because from these behaviours comes an increased possibility of compliance, as well as a good technical approach;

• Prescription: this medical act must be complete, otherwise on the side of physician (MD) there is not enough knowledge to be part of the team, to follow accurately the subsequent steps, and finally to treat adequately through bracing;

• Construction: this therapeutic act by the orthotist (CPO) must follow certain steps to allow a proper development of the brace;

• Brace check: this is an unavoidable, highly important step in brace treatment; it is the verification that the interaction between the brace and the body and pathology correspond to what has been planned during prescription and built during construction;

• Follow-up: bracing does not finish with the brace check, but continues in all other medical and therapeutic acts, that must follow considering continuously the brace as the mainstay of all what is done on and with the patient.

Following we report and discuss each single recommendation.

### Recommendation 1

The MD responsible for the treatment has to be experienced and should fulfil all these requirements:

1. training by a previous master (i.e. MD with at least 5 years of experience in bracing) for at least 2 years

2. at least 2 years of continuous practice in scoliosis bracing

3. prescription of at least 1 brace per working week (~45 per year) in the last 2 years

4. evaluation of at least 4 scoliosis patients per working week (~150 per year) in the last 2 years

Due to the actual situation of conservative treatment in many countries, this must be considered the ideal to be reached as soon as possible through education. Nevertheless, it must be recognised that experience and preparation is the only way to avoid problems to patients and reach adequate results in this field.

This recommendation has to be applied in everyday clinics and in research on clinical efficacy of bracing.

#### Discussion

This recommendation has been developed with the aim of stating the need for continuous experience and training for MDs prescribing braces. There was the strong feeling on the need of introducing some numbers to better define the recommendation. The more experienced on bracing strongly proposed higher numbers than those finally decided (and this was also statistically significantly different). In the meantime there was some fear of being excluded on the side of those already working in the field, but without big numbers of patients treated. This led to a final accepted compromise. [, and to the general comment that conclude the recommendation and comes from the reality around the world.] In the meantime SOSORT is ready to give courses on bracing, as well as in supporting people who do need to increase their knowledge in this field through adequate masterships.

### Recommendation 2

The CPO constructing braces has to be experienced and should fulfil all these requirements

1. working continuously with a master MD (i.e. a MD fulfilling to recommendation 1 criteria) for at least 2 years

2. at least 2 years of continuous practice in scoliosis bracing

3. construction of at least 2 braces per working week (~100 per year) in the last 2 years

Due to the actual situation of conservative treatment in many countries, this must be considered the ideal to be reached as soon as possible through education. Nevertheless, it must be recognised that experience and preparation is the only way to avoid problems to patients and reach adequate results in this field

This recommendation has to be applied in everyday clinics and in research on clinical efficacy of bracing.

#### Discussion

This recommendation is the twin of the previous one on the practical side, and the same considerations can be applied.

### Recommendation 3

To ensure optimum results, the MD, CPO and physiotherapist (PT) must work together as a multiprofessional team. This can be accomplished, even if they are not currently located in the same workplace, through continuous exchange of information, team meetings, and verification of braces in front of single patients

This recommendation has to be applied in clinics and research.

#### Discussion

The importance of a team approach in bracing management of scoliosis clearly emerged from the discussion in Athens and is underlined in the this and other recommendations. It has already been proven since many years the importance of such an approach in rehabilitation [33], because the patient's active and informed participation is required to obtain results. In this context we have to deal with children and adolescents having to wear plastics for many hours per day, for years; the main problem is compliance [30,34-37]. Compliance is not a problem of the kind of treatment, per se, but is a problem of management [3], an MDs' and team's problem. According to the SOSORT Consensus, a team approach is the best solution for compliance achievement and maintenance. Together with a team approach, all the recommendations give individual clinical methodological tools to achieve the best clinical efficacy in bracing and should be strictly followed to achieve the best results.

### Recommendation 4

Commitment, time and counselling to increase compliance: MDs, CPOs and PTs have to give thorough advice and counselling to each single patient and family each time it is needed (at each contact for MDs and CPOs) provided they give as a team the same messages previously agreed

This recommendation has to be applied in everyday clinics and in research on clinical efficacy of bracing.

#### Discussion

Compliance is not related only to treatment, but also to the treating team. Commitment is crucial, because the verbal and nonverbal communication to patients and family comes directly from our beliefs. Time is another determinant element, because without time it is impossible to listen, to understand the feelings and fears of patient and family, to answer questions properly, and to give the correct explanations that will prevent problems and correct behaviours. Communication is critical, and each professional must act according to his/her knowledge. Obviously behaviours will be different according to professions: while PTs have a continuous contact with the patient (in this way with a big responsibility in allowing a proper bracing), MD and CPOs have less frequent but more intense contacts that must be particularly focused and straightforward. Finally, team behaviour is critical, because different proposals increase confusion and lead to reduced compliance, while different wording for the same proposal amplifies the message and fosters compliance.

### Recommendation 5

All the phases of brace construction have to be followed for each single brace

1. prescription by a well trained and experienced MD (fulfilling recommendation 1 criteria)

2. construction by a well trained and experienced CPO (fulfilling recommendation 2 criteria)

3. check by the MD in team with the CPO, and possibly the PT

4. correction by the CPO according to MD indications

5. follow-up by the CPO, MD and PT

This recommendation has to be applied in everyday clinics and in research on clinical efficacy of bracing.

#### Discussion

Bracing can be compared to the check of a building by engineers. All phases related to bracing can in fact be compared to an engineering act: planning (prescription), building (construction), testing (check). Here we also have the possibility/need of correction of the brace according to the check; corrections are required almost always, because a discrepancy between the project and the interaction between the brace and the body of the patient is very common.

The treating team is obviously multidisciplinary, but there are roles that must be maintained and phases that must be followed. In bracing most of the direct work relies on MDs and CPOs; nevertheless, the impact of the entire team, including the PT, to increase compliance is underlined here.

### Recommendation 6

In each single prescription of a brace (case by case), the MD must:

1. write the details of brace construction (where to push and where to leave space, how to act on the trunk to obtain results on the spine) when not already defined "a priori" with the CPO

2. prescribe the exact number of hours of brace wearing

3. be totally convinced of the brace proposed and committed to the treatment

4. use any ethical means to increase patient compliance, including thorough explanation of the treatment, aids such as photos, brochures, video, etc

This recommendation has to be applied in everyday clinics and in research on clinical efficacy of bracing.

#### Discussion

Prescription is the start of bracing, and a good start is crucial. If the MD has a classification from which the brace derives, it is perhaps enough to state the classification in the prescription to give all details to the CPO. But the MD takes the responsibility of this first stage, and he must state everything adequately for the subsequent stages. This cannot be delegated to the CPO, otherwise the patient will lack the MD role in the treating team.

Conviction and commitment is underlined here because the MD has the maximum importance in the eyes of patient and family, and is continuously regarded as the leader and master of treatment: his behaviour is crucial.

The ways to increase compliance should be used, but it is also underlined the importance of avoiding unethical behaviour. Invoking catastrophic ideas to increase compliance can create lifelong problems to the patient and family. We, in SOSORT, are aware of such behaviours and we strongly condemn such psychological terrorism.

### Recommendation 7

In each single construction of a brace, case by case, the CPO has to:

1. check the prescription and its details and eventually discuss them with the prescribing MD, if needed, before construction

2. fully execute the agreed prescription

3. be totally convinced of the brace proposed and committed to the treatment

4. use any ethical means to increase patient compliance, including thorough explanation of the treatment, aids such as photos, brochures, video, etc

This recommendation has to be applied in clinics and research.

#### Discussion

This recommendation is the twin of the previous one on the practical side, and the same considerations can be applied. The construction of the brace must follow the MD prescription. Obviously, in a team there can be discussion. The CPO has the responsibility to discuss if he does not agree on the MD's indications, even if the responsibility of the brace is on the shoulders of the MD. The CPO takes the responsibility of the stage of construction, otherwise we lack the CPO role in the treating team.

### Recommendation 8

In each single check of a brace, case by case, the responsible MD in partnership with the CPO has to:

1. verify accurately if it fits properly and fulfils the needs of the individual patient

2. check the scoliosis correction in all three planes (frontal, sagittal and horizontal)

3. check clinically the aesthetic correction

4. maximize brace tolerability (reduce visibility and allow movements and activity of daily life as much as possible for the chosen technique)

5. apply all changes needed and, if necessary, even rebuild the brace without extra-charge for patients

6. check the corrections applied

7. check that the patient (and/or his/her parents) is able to apply or put on the brace properly

8. access the patient's mood and counsel him and the family at brace delivery and at other follow-ups

This recommendation has to be applied in clinics and research.

#### Discussion

Bracing is like an engineering project, but the project is not always totally correct and must be checked in the reality. This check must be done both by the MD on the side of the project, and the CPO on the side of the technical application. The lack of check does not allow a proper brace to be applied. In this check both technical (points 1, 2, 3, 5, 6, 7) and compliance (points 1, 4, 7, 8) aspects must be carefully considered. These points go beyond the technical approach used. Obviously some braces fulfil better some points when compared to others, but all techniques should fulfil all these points as much as possible. Unbalancing toward efficacy while not respecting acceptability will lead to bad compliance and failure. Conversely, increasing acceptability while decreasing efficacy will lead to failure as well.

### Recommendation 9

The check of each single brace has to be clinical and/or radiographic

This recommendation has to be applied in clinics and research.

#### Discussion

On this point there was discussion because the different schools do not agree on how to check the single braces. Some strongly support a radiographic check, while others check only clinically to reduce the burden of radiation exposure [38]. In this respect also social differences in different countries can play a role, because there are nations where repeated radiographs are considered almost unacceptable to growing children and adolescents. Even in this case it is very important to develop careful clinical evaluations to check the brace

### Recommendation 10

The MD, CPO and PT must check the brace and patient compliance regularly (MDs and CPOs each time they see the patient), and reinforce the usefulness of brace treatment to the patient and his/her family.

This recommendation has to be applied in clinics and research.

#### Discussion

This recommendation is implicit in previous sections, but we underline once again the importance of each professional in creating, strengthening and maintaining compliance.

### Recommendation 11

The MD has to follow-up the braced patient regularly, at least every 3 to 6 months. Standard intervals have to be reduced according to individual needs (first brace, growth spurt, progressive or atypical curve, poor compliance, request of other team members – CPO, PT ...). Using tools (written protocols, recalls...) to keep patients informed of their follow-up is strongly suggested.

This recommendation has to be applied in clinics and research.

#### Discussion

MDs have to plan meetings with the patients according to medical but also therapeutic needs, and adapt to the individual needs. Sometimes the patient is not aware of the importance of these meetings. For practical and/or cost reasons they may minimize their meetings with the MD, unduly creating management problems that strongly reflect on the efficacy of treatment. In this respect tools are suggested to make patients aware of their role.

### Recommendation 12

The brace has to be changed for a new one as soon as the child grows up or the brace loses efficacy, and this need can be suggested by the CPO, but is the responsibility of the treating MD

This recommendation has to be applied in clinics and research.

#### Discussion

Children and adolescents grow up, while plastic remains the same. This clearly evident statement sometimes is not so evident to MDs and/or health systems and/or patients itself where the main point is reducing costs. nce carefully considered on the side of MDs to construct braces that can have some adaptability to bodily changes so following the patients' growth, it is also important to state the role of payers in supporting a correct treatment.] It is also underlined that the responsibility of the MD must be considered also in this case, while remembering that in most of the cases he can act as a third party [in front of] the CPO (that usually earns money from the brace construction, while the MD does not).

### Recommendation 13

The CPO has to regularly check the brace. In front of any problem, he/she has to refer to the treating MD

This recommendation has to be applied in everyday clinics and in research on clinical efficacy of bracing.

#### Discussion

This point is crucial. Obviously different braces will require different maintenance. In addition, the CPO behaviour can vary in different treating teams, where some compliance corrections could be left to the CPO. In any case any correction that interferes with the technical correction of the curve must be seen by the MD, who bears responsibility for treatment.

### Recommendation 14

The PT has to check the brace regularly. In response to any problem, she/he has to refer to the MD and not to the patient. As a member of the treating team, he/she has to be trained to face the problems of compliance, or the needs for more explanation by the patient or his/her family. In case she/he is not entirely a member of the treating team he must not act autonomously and must refer to the treating MD.

This recommendation has to be applied in clinics and research.

#### Discussion

PTs interact with the patient on a regular basis, as much as twice or three times per week. In this way PTs have a continuous and determinant role throughout all scoliosis treatment. They must on one side understand their role. Sometimes this role is to serve as an interface among the patient and MDs and CPOs; nevertheless they must be totally unbalanced toward efficacy of treatment, that rely on the different responsibilities of MDs and CPOs as well. Consequently, on one side they must reinforce compliance, on the other they must develop their knowledge on bracing so to be able to make some checks regularly and pose questions to CPOs and MDs to increase the efficacy and tolerability of individual braces for the sake of the patient. In this recommendation it is also strongly suggested that PTs have to refer to the other members of the treating team and not directly to the patients and families. Confusion and mixed messages from team members lead to bad compliance.

## Conclusion

The last SOSORT Consensus here presented give strong recommendations on the management of scoliosis through bracing. Key elements of the recommendations are efficacy as well as compliance. The latter stems from the treatment proposed, as well as responses of the patient and family, and is strictly correlated also to behaviours of the treating team. Bracing success is higher inside SOSORT [8-12] than what is generally reported in the literature [7]. That this difference is not due to technical reasons only is evident from a previous Consensus in which we failed to show strong similarities in the technical factor among SOSORT members. On the contrary we reached an high degree of Consensus on management criteria, that are now openly proposed to the general community (Additional file 5) both for clinical everyday use and research on clinical efficacy of bracing.

## Competing interests

The authors declare that they have no competing interests.

## Authors' contributions

All the authors have made substantial contributions to conception, design, and interpretation of data; have been involved in revising critically the manuscript for important intellectual content; have given final approval of the version to be published. SN and FZ have been involved in the acquisition and analysis of data. SN provided the drafting of the manuscript.

## Supplementary Material

Additional file 1 **First draft of recommendations**. First draft of recommendations. Click here for file

Additional file 2 **Pre-Meeting Questionnaire**. Pre-Meeting Questionnaire. Click here for file

Additional file 3 **Meeting Questionnaire**. Meeting Questionnaire. Click here for file

Additional file 4 **Standards of management of idiopathic scoliosis with corrective braces in everyday clinics and in clinical research. The SOSORT Criteria for bracing**. Standards of management of idiopathic scoliosis with corrective braces in everyday clinics and in clinical research. The SOSORT Criteria for bracing. Click here for file

Additional file 5 **Questionnaire to verify the achievement of the SOSORT Criteria for bracing: "Standards of management of idiopathic scoliosis with corrective braces in everyday clinics and in clinical research"**. Questionnaire to verify the achievement of the SOSORT Criteria for bracing: "Standards of management of idiopathic scoliosis with corrective braces in everyday clinics and in clinical research". This is now openly proposed to the general community both for clinical everyday use and research on clinical efficacy of bracing. Click here for file
