# Supplementary material for: Guidelines on "Standards of management of idiopathic scoliosis with corrective braces in everyday clinics and in clinical research": SOSORT Consensus 2008
Source: Scoliosis. 2009 Jan 16;4:2. doi: 10.1186/1748-7161-4-2 (PMC2651850; doi:10.1186/1748-7161-4-2)
Supplement: Additional file 1 — First draft of recommendations. First draft of recommendations. [file 1748-7161-4-2-S1.doc]

# SOSORT Consensus Paper 2008

In this paper you will find (1) the methods of Consensus (that will remain here and eventually be amended according to any need); and (2) the recommendations.

## What you have to do now (if you want):

Please, **read** the Recommendations that start in the next page and feel free to **make any change** you want; if you want, **add** any more recommendation you think that could be useful.

Then **send** everything to me: I will collate everything in a single set of recommendations and send it back to you for your final acceptance. Then, I will send them to all SOSORT members for ranking.

**Please, consider that these recommendations derive from my own experience and that of my friends and colleagues in Italy. In any case, I have been careful to propose something that could be generally agreed**. For example, in the last recommendations I stated that braces should be checked clinically AND radiographically; even if I do not follow this approach; nevertheless I understand that almost all people do it, and that perhaps I must change my habits. **Perhaps there are recommendations that you do not agree on. Please state it clearly, because we need to find an agreement first of all among ourselves, if we want to propose something general, meaningful and useful.**  In proposing changes or new recommendations, please, try to **remain general and not too individual**.

# Group 1: experience

## Recommendation n.1

The **physician** prescribing braces must be **experienced** and he should fulfil all these requirements:

- training by a previous master for some years
- at least 5 years of continuous practice in scoliosis bracing
- prescription of at least 100 braces per year (2.5 per working week)
- evaluation of at least 200 scoliosis patients per year (5 per working week)

### Comments

- - - 1. This recommendation has been developed with the aim of stating the need for continuous experience and training for physicians prescribing braces: feel free to propose other indicators or change the numbers above. I think that we need some numbers, otherwise the idea of experience is too fable if not quantified

## Recommendation n.2

The **orthotist** constructing braces must be **experienced** and he should fulfil all these requirements:

- working continuously with a master physician for some years
- at least 5 years of continuous practice in scoliosis bracing
- construction of at least 100 braces per year (2.5 per working week)

### Comments

1. This recommendation has been developed with the aim of stating the need for continuous experience and training for orthotists constructing braces: feel free to propose other indicators or change the numbers above. I think that we need some numbers, otherwise the idea of experience is too fable if not quantified

# Group 2: behaviours

## Recommendation n.3

To ensure the maximum results, the physician and the orthotist must work together as a **multiprofessional team**

### Comments

1. The recommendation has been proposed because the idea of team is crucial in this field to have same language both for technical and human (i.e. patient compliance) reasons

## Recommendation n.4

**Commitment, time and speeches to increase compliance**: both physician and orthotist must give thorough advice and counselling to each single patient and family each time they see them

### Comments

1. Compliance is a key factor of the results we obtained and, to me, without fulfilling this recommendation the risk is to fail everything

## Recommendation n.5

All the phases of **brace construction** must be followed for each single brace:

- prescription by a well trained and experienced physician
- construction by a well trained and experienced orthotist
- check by the physician in team with the orthotist
- corrected by the orthotist according to physician indications

### Comments

1. The treating team is multidisciplinar, but there are roles that must be maintained and phases that must be followed.

# Group 3: brace prescription

## Recommendation n.6

In each single **prescription of a brace** (case by case), the **physician** must:

- chose the type of brace
- write the details of brace construction needed
- prescribe the exact number of hours of brace wearing
- be totally convinced of the brace proposed and committed to the treatment
- use any mean to increase patient compliance, including through explanation of the treatment, aids such as photos, brochures, video, etc

### Comments

1. Prescription is the start of everything, and a good start is crucial. Obviously, if the physician has a classification from which the brace derives, it’s perhaps enough to state the classification in the prescription. But the physician take the responsibility of this first stage, and he must state everything adequately for the subsequent stages. This cannot be delegated to the orthotist, otherwise we lack the physician role.

# Group 4: brace construction

## Recommendation n.7

In each single **construction of a brace** (case by case), the **orthotist** must:

- check the prescription and its details and eventually discuss them with the prescribing physician, if needed
- fully execute the prescription
- be totally convinced of the brace proposed and committed to the treatment
- use any mean to increase patient compliance, including through explanation of the treatment, aids such as photos, brochures, video, etc

### Comments

1. The construction of the brace must follow the physician prescription. Obviously, in a team there can be discussion, that is the richness of a team, and this is considered. The orthotist anyway take the responsibility of this stage, otherwise we lack the orthotist role.

# Group 5: brace check

## Recommendation n.8

In each single **check of a brace** (case by case), the **physician and orthotist** must:

- verify accurately if it fulfils the need of the individual patient
- check the scoliosis correction in all the three planes (frontal, sagittal and horizontal)
- check the aesthetic correction
- maximize brace tolerability
- check that the patient can perform movements and activity of daily life as much as possible
- require any change needed

### Comments

1. Prescription is like a project, but the project is not always totally correct and must be checked in the reality. This check must be done both by the physician on the side of the project, and the orthotist on the side of the technical application. The lack of check do not allow a proper brace to be applied. In this check I listed both technical and compliance aspects. I do understand that not all of us have the same feelings, but I think that the way everything is written here could be accepted. Nevertheless, please propose any change you want.

## Recommendation n.9

The check of each single brace must be **clinical and/or radiographic**

### Comments

1. I wait for your comments here.
