# Supplementary material for: Guidelines on "Standards of management of idiopathic scoliosis with corrective braces in everyday clinics and in clinical research": SOSORT Consensus 2008
Source: Scoliosis. 2009 Jan 16;4:2. doi: 10.1186/1748-7161-4-2 (PMC2651850; doi:10.1186/1748-7161-4-2)
Supplement: Additional file 2 — Pre-Meeting Questionnaire. Pre-Meeting Questionnaire. [file 1748-7161-4-2-S2.doc]

# TO: SOSORT Current and Former Members RE: Invitation to participate in our Consensus for 2008

Dear SOSORT Member,

You can find attached (1) Instructions for taking part in our Consensus for 2008; (2) The fourteen recommendations proposed for SOSORT Consensus Paper 2008, and (3) The topic chosen at our meeting in Boston, 2007.

The time required for completion of the documentation is short but critical to our goals. Your participation in this work is very important for our Society as well as for our future work, and to strengthen future publications in this field. Your work will be recognised as previously done in SOSORT Consensus papers, by listing your names in the final publication.

Best regards

Stefano Negrini

Coordinator of SOSORT Consensus 2008

## Instructions

## (the questionnaire is apparently long, but the questions for the 14 recommendations - 1 per page - are similar)

## (You will not spend too much time, and you will contribute a lot to SOSORT)

Please, **read** the 14 Recommendations (in yellow in the text) that start in the next pages and then rank your preferences (they are similar throughout the entire document):

**tick the appropriate choices** in the scales proposed:

- agreement,
- importance,
- applicability

**Add any comment** you want, and **propose any change**, if you want, but **explain** your position in the notes. Then **send** everything to me by e-mail

**DEADLINE: 31 January 2008**

# Your data (to be inserted in the paper, if you wish)

- If you do not want your name inserted in the paper, thick here and omit your name but, for statistical reasons, **compile in any case the anagraphical and professional information**

First and family name: _____________________________________________________________

Gender, age: _____________________________________________________________________

Profession: ______________________________________________________________________

Medical specialty (for MDs): ________________________________________________________

Number of braces prescribed and/or constructed and/or checked per year: _____________________

Number of year of experience in the field of scoliosis: ____________________________________

# Group 1: Experience / competence

## Recommendation #1

The **physician** responsible for the treatment has to be **experienced** and should fulfil all these requirements:

- training by a previous master (i.e. a physician with at least 10 years of experience in bracing) for at least 3 years
- at least 3 years of continuous practice in scoliosis bracing
- prescription of at least 2 braces per working week (~90 per year) in the last 2 years
- evaluation of at least 8 scoliosis patients per working week (~300 per year) in the last 2 years

### Ranking

| Agreement | Importance | Type of recommendation |
| --- | --- | --- |
| - I totally agree | - Highest | - Mandatory |
| - I agree | - High | - Highly recommended |
| - I do not agree nor disagree | - Medium | - Recommended |
| - I disagree | - Low | - Could be recommended |
| - I totally disagree | - Lowest | - Not to recommend at all |
| I apply this recommendation personally and require my team to apply it | This recommendation should be applied | |
|  | by clinicians | by researchers |
| - Always | - Always | - Always |
| - Frequently | - Frequently | - Frequently |
| - Sometimes | - Sometimes | - Sometimes |
| - Rarely | - Rarely | - Rarely |
| - Never | - Never | - Never |

### Details

#### Training by a previous master for at least 3 years

- OK as it is
- Change to: at least ____ years
- Eliminate

#### Previous master (i.e. a physician with at least 10 years of experience in bracing)

- OK as it is
- Change to: at least ____ years
- Eliminate

#### At least 3 years of continuous practice in scoliosis bracing

- OK as it is
- Change to: at least ____ years
- Eliminate

**Prescription of at least 2 braces per working week (~90 per year) in the last 2 years**

- OK as it is
- Change to: at least ____ brace per working week in the last ___ years
- Eliminate

**Evaluation of at least 8 scoliosis patients per working week (~300 per year) in the last 2 years**

- OK as it is
- Change to: at least ____ scoliosis patients per working week in the last ___ years
- Eliminate

### Details that I propose to add here

________________________________________________________________________________

________________________________________________________________________________

________________________________________________________________________________

### Comments

________________________________________________________________________________

________________________________________________________________________________

________________________________________________________________________________

## Recommendation #2

The **orthotist** constructing braces has to be **experienced** and should fulfil all these requirements:

- working continuously with a master physician (i.e. a physician fulfilling to recommendation 1 criteria) for at least 2 years
- at least 5 years of continuous practice in scoliosis bracing
- construction of at least 4 braces per working week (~200 per year) in the last 2 years

### Ranking

| Agreement | Importance | Type of recommendation |
| --- | --- | --- |
| - I totally agree | - Highest | - Mandatory |
| - I agree | - High | - Highly recommended |
| - I do not agree nor disagree | - Medium | - Recommended |
| - I disagree | - Low | - Could be recommended |
| - I totally disagree | - Lowest | - Not to recommend at all |
| I apply this recommendation personally and require my team to apply it | This recommendation should be applied | |
|  | by clinicians | by researchers |
| - Always | - Always | - Always |
| - Frequently | - Frequently | - Frequently |
| - Sometimes | - Sometimes | - Sometimes |
| - Rarely | - Rarely | - Rarely |
| - Never | - Never | - Never |

### Details

#### Working continuously with a master physician for at least 2 years

- OK as it is
- Change to: at least ____ years
- Eliminate

#### At least 5 years of continuous practice in scoliosis bracing

- OK as it is
- Change to: at least ____ years
- Eliminate

**Construction of at least 4 braces per working week (~200 per year) in the last 2 years**

- OK as it is
- Change to: at least ____ braces per working week in the last ___ years
- Eliminate

### Details that I propose to add here

________________________________________________________________________________

________________________________________________________________________________

________________________________________________________________________________

________________________________________________________________________________

### Comments

________________________________________________________________________________

________________________________________________________________________________

________________________________________________________________________________

________________________________________________________________________________

# Group 2: Behaviours

## Recommendation #3

To ensure optimum results, the physician and the orthotist must work together as a **multiprofessional team**, through continuous exchange of information, team meetings, and verification of braces in front of single patients.

### Ranking

| Agreement | Importance | Type of recommendation |
| --- | --- | --- |
| - I totally agree | - Highest | - Mandatory |
| - I agree | - High | - Highly recommended |
| - I do not agree nor disagree | - Medium | - Recommended |
| - I disagree | - Low | - Could be recommended |
| - I totally disagree | - Lowest | - Not to recommend at all |
| I apply this recommendation personally and require my team to apply it | This recommendation should be applied | |
|  | by clinicians | by researchers |
| - Always | - Always | - Always |
| - Frequently | - Frequently | - Frequently |
| - Sometimes | - Sometimes | - Sometimes |
| - Rarely | - Rarely | - Rarely |
| - Never | - Never | - Never |

### Comments

________________________________________________________________________________

________________________________________________________________________________

________________________________________________________________________________

________________________________________________________________________________

## Recommendation #4

**Commitment, time and speeches to increase compliance**: Both physician and orthotist have to give thorough advice and counselling to each single patient and family each time they see them

### Ranking

| Agreement | Importance | Type of recommendation |
| --- | --- | --- |
| - I totally agree | - Highest | - Mandatory |
| - I agree | - High | - Highly recommended |
| - I do not agree nor disagree | - Medium | - Recommended |
| - I disagree | - Low | - Could be recommended |
| - I totally disagree | - Lowest | - Not to recommend at all |
| I apply this recommendation personally and require my team to apply it | This recommendation should be applied | |
|  | by clinicians | by researchers |
| - Always | - Always | - Always |
| - Frequently | - Frequently | - Frequently |
| - Sometimes | - Sometimes | - Sometimes |
| - Rarely | - Rarely | - Rarely |
| - Never | - Never | - Never |

### Comments

________________________________________________________________________________

________________________________________________________________________________

________________________________________________________________________________

________________________________________________________________________________

## Recommendation #5

All the phases of **brace construction** have to be followed for each single brace:

- prescription by a well trained and experienced physician (fulfilling recommendation 1 criteria)
- construction by a well trained and experienced orthotist (fulfilling recommendation 2 criteria)
- monitoring by the physician as a team with the orthotist, and possibly the physiotherapist
- correction by the orthotist according to physician indications
- follow-up by the orthotist, physician and physical therapist

### Ranking

| Agreement | Importance | Type of recommendation |
| --- | --- | --- |
| - I totally agree | - Highest | - Mandatory |
| - I agree | - High | - Highly recommended |
| - I do not agree nor disagree | - Medium | - Recommended |
| - I disagree | - Low | - Could be recommended |
| - I totally disagree | - Lowest | - Not to recommend at all |
| I apply this recommendation personally and require my team to apply it | This recommendation should be applied | |
|  | by clinicians | by researchers |
| - Always | - Always | - Always |
| - Frequently | - Frequently | - Frequently |
| - Sometimes | - Sometimes | - Sometimes |
| - Rarely | - Rarely | - Rarely |
| - Never | - Never | - Never |

### Details

#### Prescription by a well trained and experienced physician

- OK as it is
- Change to: ________________________
- Eliminate

**Construction by a well trained and experienced orthotist**

- OK as it is
- Change to: ________________________
- Eliminate

**Check by the physician in team with orthotist, and possibly physiotherapist**

- OK as it is
- Change to: ________________________
- Eliminate

**Correction by the orthotist according to physician indications**

- OK as it is
- Change to: ________________________
- Eliminate

**Follow-up by the orthotist, physician and physical therapist**

- OK as it is
- Change to: ________________________
- Eliminate

### Details that I propose to add here

________________________________________________________________________________

________________________________________________________________________________

________________________________________________________________________________

________________________________________________________________________________

### Comments

________________________________________________________________________________

________________________________________________________________________________

________________________________________________________________________________

________________________________________________________________________________

# Group 3: Brace prescription

## Recommendation #6

In each single **prescription of a brace** (case by case), the **physician** must:

- chose the type of brace
- write the details of brace construction (e.g. where to push and where to leave space, how to act on the trunk to obtain effects on the spine)
- prescribe the exact number of hours of brace wearing
- be totally convinced of the brace proposed and committed to the treatment
- use any means to increase patient compliance, including through explanation of the treatment, as well as aids such as photos, brochures, video, etc

### Ranking

| Agreement | Importance | Type of recommendation |
| --- | --- | --- |
| - I totally agree | - Highest | - Mandatory |
| - I agree | - High | - Highly recommended |
| - I do not agree nor disagree | - Medium | - Recommended |
| - I disagree | - Low | - Could be recommended |
| - I totally disagree | - Lowest | - Not to recommend at all |
| I apply this recommendation personally and require my team to apply it | This recommendation should be applied | |
|  | by clinicians | by researchers |
| - Always | - Always | - Always |
| - Frequently | - Frequently | - Frequently |
| - Sometimes | - Sometimes | - Sometimes |
| - Rarely | - Rarely | - Rarely |
| - Never | - Never | - Never |

### Details

#### Chose the type of brace

- OK as it is
- Change to: ________________________
- Eliminate

**Write the details of brace construction (where to push and where to leave space, how to act on the trunk to obtain results on the spine)**

- OK as it is
- Change to: ________________________
- Eliminate

**Prescribe the exact number of hours of brace wearing**

- OK as it is
- Change to: ________________________
- Eliminate

**Be totally convinced of the brace proposed and committed to the treatment**

- OK as it is
- Change to: ________________________
- Eliminate

**Use any mean to increase patient compliance, including thorough explanation of the treatment, aids such as photos, brochures, video, etc**

- OK as it is
- Change to: ________________________
- Eliminate

### Details that I propose to add here

________________________________________________________________________________

________________________________________________________________________________

### Comments

________________________________________________________________________________

________________________________________________________________________________

# Group 4: Brace construction

## Recommendation #7

In each single **construction of a brace** (case by case), the **orthotist** has to:

- check the prescription and its details and eventually discuss them with the prescribing physician, if needed
- fully execute the prescription
- be totally convinced of the brace proposed and committed to the treatment
- use any mean to increase patient compliance, including thorough explanation of the treatment, aids such as photos, brochures, video, etc

### Ranking

| Agreement | Importance | Type of recommendation |
| --- | --- | --- |
| - I totally agree | - Highest | - Mandatory |
| - I agree | - High | - Highly recommended |
| - I do not agree nor disagree | - Medium | - Recommended |
| - I disagree | - Low | - Could be recommended |
| - I totally disagree | - Lowest | - Not to recommend at all |
| I apply this recommendation personally and require my team to apply it | This recommendation should be applied | |
|  | by clinicians | by researchers |
| - Always | - Always | - Always |
| - Frequently | - Frequently | - Frequently |
| - Sometimes | - Sometimes | - Sometimes |
| - Rarely | - Rarely | - Rarely |
| - Never | - Never | - Never |

### Details

#### Check the prescription and its details and eventually discuss them with the prescribing physician, if needed

- OK as it is
- Change to: ________________________
- Eliminate

#### Fully execute the prescription

- OK as it is
- Change to: ________________________
- Eliminate

**Be totally convinced of the brace proposed and committed to the treatment**

- OK as it is
- Change to: ________________________
- Eliminate

**Use any mean to increase patient compliance, including thorough explanation of the treatment, aids such as photos, brochures, video, etc**

- OK as it is
- Change to: ________________________
- Eliminate

### Details that I propose to add here

________________________________________________________________________________

________________________________________________________________________________

________________________________________________________________________________

________________________________________________________________________________

### Comments

________________________________________________________________________________

________________________________________________________________________________

________________________________________________________________________________

________________________________________________________________________________

# Group 5: Brace check

## Recommendation #8

In each single **check of a brace** (case by case), the **physician and orthotist** has to:

- verify accurately if it fulfils the need of the individual patient
- check the scoliosis correction in all the three planes (frontal, sagittal and horizontal)
- check the aesthetic correction
- maximize brace tolerability (reduce visibility as much as possible for the used technique and allow movements and activity of daily life as much as possible)
- apply all changes needed and, if necessary, even rebuild the brace without extra-charge for patients
- check that the patient (and/or his/her parents) is able to fit the brace adequately
- verify the patients mood and counsel him and the family at brace delivery

### Ranking

| Agreement | Importance | Type of recommendation |
| --- | --- | --- |
| - I totally agree | - Highest | - Mandatory |
| - I agree | - High | - Highly recommended |
| - I do not agree nor disagree | - Medium | - Recommended |
| - I disagree | - Low | - Could be recommended |
| - I totally disagree | - Lowest | - Not to recommend at all |
| I apply this recommendation personally and require my team to apply it | This recommendation should be applied | |
|  | by clinicians | by researchers |
| - Always | - Always | - Always |
| - Frequently | - Frequently | - Frequently |
| - Sometimes | - Sometimes | - Sometimes |
| - Rarely | - Rarely | - Rarely |
| - Never | - Never | - Never |

### Details

**Verify accurately if it fulfils the need of the individual patient**

- OK as it is
- Change to: ________________________
- Eliminate

**Check the scoliosis correction in all the three planes (frontal, sagittal and horizontal)**

- OK as it is
- Change to: ________________________
- Eliminate

**Check the aesthetic correction**

- OK as it is
- Change to: ________________________
- Eliminate

**Maximize brace tolerability (reduce visibility as much as possible for the used technique and allow movements and activity of daily life as much as possible)**

- OK as it is
- Change to: ________________________
- Eliminate

**Apply all changes needed and, if necessary, even rebuild the brace without extra-charge for patients**

- OK as it is
- Change to: ________________________
- Eliminate

**Check that the patient (and/or his/her parents) is able to fit the brace adequately**

- OK as it is
- Change to: ________________________
- Eliminate

**Verify the patients mood and counsel him and the family at brace delivery**

- OK as it is
- Change to: ________________________
- Eliminate

### Details that I propose to add here

________________________________________________________________________________

________________________________________________________________________________

________________________________________________________________________________

________________________________________________________________________________

### Comments

________________________________________________________________________________

________________________________________________________________________________

________________________________________________________________________________

________________________________________________________________________________

## Recommendation #9

The check of each single brace has to be **clinical and/or radiographic**

### Ranking

| Agreement | Importance | Type of recommendation |
| --- | --- | --- |
| - I totally agree | - Highest | - Mandatory |
| - I agree | - High | - Highly recommended |
| - I do not agree nor disagree | - Medium | - Recommended |
| - I disagree | - Low | - Could be recommended |
| - I totally disagree | - Lowest | - Not to recommend at all |
| I apply this recommendation personally and require my team to apply it | This recommendation should be applied | |
|  | by clinicians | by researchers |
| - Always | - Always | - Always |
| - Frequently | - Frequently | - Frequently |
| - Sometimes | - Sometimes | - Sometimes |
| - Rarely | - Rarely | - Rarely |
| - Never | - Never | - Never |

### Comments

________________________________________________________________________________

________________________________________________________________________________

________________________________________________________________________________

________________________________________________________________________________

# Group 6: Brace follow-up

## Recommendation #10

The **physician, orthotist and physical therapist** must check the brace each time they see the patient, as well as check carefully patient compliance and reinforce the usefulness of brace treatment to the patient and his/her family.

### Ranking

| Agreement | Importance | Type of recommendation |
| --- | --- | --- |
| - I totally agree | - Highest | - Mandatory |
| - I agree | - High | - Highly recommended |
| - I do not agree nor disagree | - Medium | - Recommended |
| - I disagree | - Low | - Could be recommended |
| - I totally disagree | - Lowest | - Not to recommend at all |
| I apply this recommendation personally and require my team to apply it | This recommendation should be applied | |
|  | by clinicians | by researchers |
| - Always | - Always | - Always |
| - Frequently | - Frequently | - Frequently |
| - Sometimes | - Sometimes | - Sometimes |
| - Rarely | - Rarely | - Rarely |
| - Never | - Never | - Never |

### Comments

________________________________________________________________________________

________________________________________________________________________________

________________________________________________________________________________

________________________________________________________________________________

## Recommendation #11

The **physician** has to follow-up the efficacy of the brace at least every 4 to 6 months, and eventually before if required by the orthotist and/or the physical therapist.

### Ranking

| Agreement | Importance | Type of recommendation |
| --- | --- | --- |
| - I totally agree | - Highest | - Mandatory |
| - I agree | - High | - Highly recommended |
| - I do not agree nor disagree | - Medium | - Recommended |
| - I disagree | - Low | - Could be recommended |
| - I totally disagree | - Lowest | - Not to recommend at all |
| I apply this recommendation personally and require my team to apply it | This recommendation should be applied | |
|  | by clinicians | by researchers |
| - Always | - Always | - Always |
| - Frequently | - Frequently | - Frequently |
| - Sometimes | - Sometimes | - Sometimes |
| - Rarely | - Rarely | - Rarely |
| - Never | - Never | - Never |

### Comments

________________________________________________________________________________

________________________________________________________________________________

________________________________________________________________________________

________________________________________________________________________________

## Recommendation #12

The brace has to be changed for a new one as soon as the child grows up, and this must be judged by the physician responsible for the treatment. Usually braces have to be changed every 12-24 months according to the growth phase, but even faster sometimes during the pubertal growth spurt.

### Ranking

| Agreement | Importance | Type of recommendation |
| --- | --- | --- |
| - I totally agree | - Highest | - Mandatory |
| - I agree | - High | - Highly recommended |
| - I do not agree nor disagree | - Medium | - Recommended |
| - I disagree | - Low | - Could be recommended |
| - I totally disagree | - Lowest | - Not to recommend at all |
| I apply this recommendation personally and require my team to apply it | This recommendation should be applied | |
|  | by clinicians | by researchers |
| - Always | - Always | - Always |
| - Frequently | - Frequently | - Frequently |
| - Sometimes | - Sometimes | - Sometimes |
| - Rarely | - Rarely | - Rarely |
| - Never | - Never | - Never |

### Comments

________________________________________________________________________________

________________________________________________________________________________

________________________________________________________________________________

________________________________________________________________________________

## Recommendation #13

The **orthotist** has to check the fitting and comfort of the brace at least every 2-3 months, or even more frequently if required by the type of brace or by the patient. In case of any doubt as to efficacy, the physician must be contacted.

### Ranking

| Agreement | Importance | Type of recommendation |
| --- | --- | --- |
| - I totally agree | - Highest | - Mandatory |
| - I agree | - High | - Highly recommended |
| - I do not agree nor disagree | - Medium | - Recommended |
| - I disagree | - Low | - Could be recommended |
| - I totally disagree | - Lowest | - Not to recommend at all |
| I apply this recommendation personally and require my team to apply it | This recommendation should be applied | |
|  | by clinicians | by researchers |
| - Always | - Always | - Always |
| - Frequently | - Frequently | - Frequently |
| - Sometimes | - Sometimes | - Sometimes |
| - Rarely | - Rarely | - Rarely |
| - Never | - Never | - Never |

### Comments

________________________________________________________________________________

________________________________________________________________________________

________________________________________________________________________________

________________________________________________________________________________

## Recommendation #14

The **physical therapist** has to check the brace each time he/she sees the patient to propose the exercises needed for treatment. This means that she/he can identify problems of wearability or efficacy before the other members of the team and refer to the physician or orthotist respectively. If there are problems of compliance, or the need of more explanation to the patient or his/her family, he/she has to be trained to face all these problems and to give the correct answers. She/he is a member of the treating team, and he/she, as well as all professionals involved, has to behave accordingly.

### Ranking

| Agreement | Importance | Type of recommendation |
| --- | --- | --- |
| - I totally agree | - Highest | - Mandatory |
| - I agree | - High | - Highly recommended |
| - I do not agree nor disagree | - Medium | - Recommended |
| - I disagree | - Low | - Could be recommended |
| - I totally disagree | - Lowest | - Not to recommend at all |
| I apply this recommendation personally and require my team to apply it | This recommendation should be applied | |
|  | by clinicians | by researchers |
| - Always | - Always | - Always |
| - Frequently | - Frequently | - Frequently |
| - Sometimes | - Sometimes | - Sometimes |
| - Rarely | - Rarely | - Rarely |
| - Never | - Never | - Never |

### Comments

________________________________________________________________________________

________________________________________________________________________________

________________________________________________________________________________

________________________________________________________________________________

# Points to be discussed at the Meeting

At this stage it is still possible to insert points to be discussed at the Meeting and considered for insertion in the final papers after voting at the Meeting itself. Here are some examples already proposed. Feel free to add any other point at the end of the document

Group 1: expertise and competence

1. Institutions/companies engaged in brace treatment of idiopathic scoliosis should apply to the national/international organism (responsible for excellence of practice) for a certificate of the appropriate level of the patients’ care/of recommendation.
2. Professionals of brace treatment should be obliged to collect the points of the continuous medical education (in a 3-year cycle).
3. ______________________________________________________________________________________________________________________________________________________________________________________________________________________________
4. ______________________________________________________________________________________________________________________________________________________________________________________________________________________________

Group 2: behaviours

1. ______________________________________________________________________________________________________________________________________________________________________________________________________________________________
2. ______________________________________________________________________________________________________________________________________________________________________________________________________________________________

Group 3: brace prescription

1. ______________________________________________________________________________________________________________________________________________________________________________________________________________________________
2. ______________________________________________________________________________________________________________________________________________________________________________________________________________________________

Group 4: brace construction

1. The shoulder girdle should be balanced in brace.
2. The single scoliosis should be overcorrected in the coronal plane when being in brace (imbalance to the opposite side should be present).
3. ______________________________________________________________________________________________________________________________________________________________________________________________________________________________
4. ______________________________________________________________________________________________________________________________________________________________________________________________________________________________

Group 5: brace check

1. The first brace should be fitted during a short in-patient stay in a specialized department.
2. The in-brace exercises are learned by the child at the consultation when the brace is fitted.
3. Psychological support is desirable during the first weeks of brace treatment.
4. ______________________________________________________________________________________________________________________________________________________________________________________________________________________________
5. ______________________________________________________________________________________________________________________________________________________________________________________________________________________________

Group 6: brace follow-up

1. Hours of daily wear are noted and presented to the physician at the next consultation.
2. Exercises should be checked at each consultation for their correctness.
3. Documentation is clinical (Bunnell), photos, surface topography, radiography.
4. Complications of brace treatment should be noted (/reported to national central database) – compression of the neurovascular bundle in the axilla, compression of the lateral femoral cutaneous nerve, skin pressure sores, etc.
5. ______________________________________________________________________________________________________________________________________________________________________________________________________________________________
6. ______________________________________________________________________________________________________________________________________________________________________________________________________________________________

Group 7 (?): daily life with the brace

1. All sports are allowed when managing a scoliotic child with a brace.
2. The sports are practiced with the brace on, except for..
3. The child may be exempted from the school sport, if wearing the brace to school.
4. ______________________________________________________________________________________________________________________________________________________________________________________________________________________________
5. ______________________________________________________________________________________________________________________________________________________________________________________________________________________________

Others

1. ______________________________________________________________________________________________________________________________________________________________________________________________________________________________
2. ______________________________________________________________________________________________________________________________________________________________________________________________________________________________
