# Supplementary material for: Guidelines on "Standards of management of idiopathic scoliosis with corrective braces in everyday clinics and in clinical research": SOSORT Consensus 2008
Source: Scoliosis. 2009 Jan 16;4:2. doi: 10.1186/1748-7161-4-2 (PMC2651850; doi:10.1186/1748-7161-4-2)
Supplement: Additional file 3 — Meeting Questionnaire. Meeting Questionnaire. [file 1748-7161-4-2-S3.doc]

# SOSORT Consensus 2008

# Standards of management of idiopathic scoliosis with corrective braces in everyday clinics and in clinical research

## **All Meeting participants** are kindly invited to give their contribution at the SOSORT Consensus on **Friday April 4^th^, 16.45 – 17.45**

## You can vote only if

- you are a **MEMBER OF SOSORT** (**in case you are not**, you can become a member immediately: please ask to the Meeting Secretary)
- and you **PARTICIPATE AT THE CONSENSUS SESSION** on **Friday April 4^th^, 16.45 – 17.45**

## Methods

If 90% of SOSORT Members already agreed in the previous round of this Delfi process, we will maintain the recommendation

- We will vote only for recommendations that did not reach 90% agreement
- We will vote also for general statements proposed by responders to the questionnaire

### Vote of agreement

Only for recommendation (or part of the recommendation) that did not reach 90% previously

- 90% or more of yes: the recommendation (or its part) will be maintained
- 89% or less of yes: the recommendation (or its part) will be deleted

### Vote of research application

Do you think that during research on brace treatment of scoliosis patients, the clinicians applying braces should follow these recommendations? Do you agree **that research in this field, to really verify brace efficacy, should be based on high quality brace treatment ?**

Obviously, according to the design of each single research, some researchers will be perhaps blinded and independent from the treating team.

#### Votes

Only for recommendations that did not reach 90% previously

- 90% or more of yes: the recommendation will be applied for future researches in this field
- 89% or less of yes: the recommendation will not be applied for future researches in this field

### Vote on changes proposed

We will vote all change? proposed in previous rounds by SOSORT members to each recommendation as a whole

- 90% or more of yes: the recommendation will be changed according to suggestion received
- 89% or less of yes: the recommendation will remain in its original form

# Recommendation 1

## Old version

The physician responsible for the treatment has to be experienced and should fulfil all these requirements:

1. training by a previous master (i.e. a physician with at least 10 years of experience in bracing) for at least 3 years
2. at least 3 years of continuous practice in scoliosis bracing
3. prescription of at least 2 braces per working week (~90 per year) in the last 2 years
4. evaluation of at least 8 scoliosis patients per working week (~300 per year) in the last 2 years

## New version

The physician responsible for the treatment has to be experienced and should fulfil all these requirements:

1. training by a previous master (i.e. a physician with at least 5 years of experience in bracing) for at least 2 years
2. at least 2 years of continuous practice in scoliosis bracing
3. prescription of at least 1 brace per working week (~45 per year) in the last 2 years
4. evaluation of at least 4 scoliosis patients per working week (~150 per year) in the last 2 years

## General statement

Due to the actual situation of conservative treatment in many countries, this must be considered the ideal to be reached as soon as possible through education. Nevertheless, it must be recognised that experience and preparation is the only way to avoid problems to patients and reach adequate results in this field

## Votes

1. Do you agree with recommendation #1 ?

- Yes
- No

2. Do you think that recommendation #1 should be applied by clinicians engaged in future research in this field ?

- Yes
- No

3. Do you agree with the general statement proposed after recommendation #1 ?

- Yes
- No

# Recommendation 2

## Old version

The orthotist constructing braces has to be experienced and should fulfil all these requirements

1. working continuously with a master physician (i.e. a physician fulfilling to recommendation 1 criteria) for at least 2 years
2. at least 5 years of continuous practice in scoliosis bracing
3. construction of at least 4 braces per working week (~200 per year) in the last 2 years

## New version

The orthotist constructing braces has to be experienced and should fulfil all these requirements

1. working continuously with a master physician (i.e. a physician fulfilling to recommendation 1 criteria) for at least 2 years
2. at least 2 years of continuous practice in scoliosis bracing
3. construction of at least 2 braces per working week (~100 per year) in the last 2 years

## General statement

Due to the actual situation of conservative treatment in many countries, this must be considered the ideal to be reached as soon as possible through education. Nevertheless, it must be recognised that experience and preparation is the only way to avoid problems to patients and reach adequate results in this field

## Votes

4. Do you agree with recommendation #2 ?

- Yes
- No

5. Do you think that recommendation #2 should be applied by clinicians engaged in future research in this field ?

- Yes
- No

6. Do you agree with the general statement proposed after recommendation #2 ?

- Yes
- No

# Recommendation 3

## Old version

To ensure the maximum results, the physician and the orthotist have to work together as a multiprofessional team, through continuous exchange of information, team meetings, and verification of braces in front of single patients

## New version

To ensure the maximum results, the physician, orthotist and physiotherapist have to work together as a multiprofessional team, even if they are not currently located in the same working place, through continuous exchange of information, team meetings, and verification of braces in front of single patients

## Votes

7. Do you think that recommendation #3 should be applied by clinicians engaged in future research in this field ?

- Yes
- No

8. Do you agree on all changes proposed to recommendation #3 ?

- Yes
- No

# Recommendation 4

## Old version

Commitment, time and speeches to increase compliance: both physician and orthotist have to give thorough advice and counselling to each single patient and family each time they see them

## New version

Commitment, time and speeches to increase compliance: physicians, orthotists and physiotherapists have to give thorough advice and counselling to each single patient and family each time it is needed (at each contact for physicians and orthotists) provided they give as a team the same messages previously agreed

## Votes

9. Do you think that recommendation #4 should be applied by clinicians engaged in future research in this field ?

- Yes
- No

10. Do you agree on all changes proposed to recommendation #4 ?

- Yes
- No

# Recommendation 5

## Old version

All the phases of brace construction have to be followed for each single brace

1. prescription by a well trained and experienced physician (fulfilling recommendation 1 criteria)
2. construction by a well trained and experienced orthotist (fulfilling recommendation 2 criteria)
3. check by the physician in team with the orthotist, and possibly the physiotherapist
4. correction by the orthotist according to physician indications
5. follow-up by the orthotist, physician and physical therapist

## New version

All the phases of brace construction have to be followed for each single brace

1. prescription by a well trained and experienced physician (fulfilling recommendation 1 criteria)
2. construction by a well trained and experienced orthotist (fulfilling recommendation 2 criteria)
3. check by the physician in team with the orthotist, and possibly the physiotherapist
4. correction by the orthotist according to physician indications
5. follow-up by the orthotist, physician and physical therapist

## Votes

11. Do you agree with point #4 of recommendation #5 ?

- Yes
- No

12. Do you agree with point #5 of recommendation #5 ?

- Yes
- No

13. Do you think that recommendation #5 should be applied by clinicians engaged in future research in this field ?

- Yes
- No

# Recommendation 6

## Old version

In each single prescription of a brace (case by case), the physician must:

1. chose the type of brace
2. write the details of brace construction (where to push and where to leave space, how to act on the trunk to obtain results on the spine)
3. prescribe the exact number of hours of brace wearing
4. be totally convinced of the brace proposed and committed to the treatment
5. use any mean to increase patient compliance, including thorough explanation of the treatment, aids such as photos, brochures, video, etc

## New version

In each single prescription of a brace (case by case), the physician must:

1. chose the type of brace
2. write the details of brace construction (where to push and where to leave space, how to act on the trunk to obtain results on the spine) when not already defined “a priori” with the CPO
3. prescribe the exact number of hours of brace wearing
4. be totally convinced of the brace proposed and committed to the treatment
5. use any ethical mean to increase patient compliance, including thorough explanation of the treatment, aids such as photos, brochures, video, etc

## Votes

14. Do you agree with point #1 of recommendation #6 ?

- Yes
- No

15. Do you agree with point #3 of recommendation #6 ?

- Yes
- No

16. Do you think that recommendation #6 should be applied by clinicians engaged in future research in this field ?

- Yes
- No

17. Do you agree on all changes proposed to recommendation #6 ?

- Yes
- No

# Recommendation 7

## Old version

In each single construction of a brace (case by case), the orthotist has to:

1. check the prescription and its details and eventually discuss them with the prescribing physician, if needed
2. fully execute the prescription
3. be totally convinced of the brace proposed and committed to the treatment
4. use any mean to increase patient compliance, including thorough explanation of the treatment, aids such as photos, brochures, video, etc

## New version

In each single construction of a brace (case by case), the orthotist has to:

1. check the prescription and its details and eventually discuss them with the prescribing physician, if needed, before construction
2. fully execute the agreed prescription
3. be totally convinced of the brace proposed and committed to the treatment
4. use any ethical mean to increase patient compliance, including thorough explanation of the treatment, aids such as photos, brochures, video, etc

## Votes

18. Do you agree with point #1 of recommendation #7 ?

- Yes
- No

19. Do you agree with point #3 of recommendation #7 ?

- Yes
- No

20. Do you agree on all changes proposed to recommendation #7 ?

- Yes
- No

# Recommendation 8

## Old version

In each single check of a brace (case by case), the physician and orthotist have to:

1. verify accurately if it fulfils the need of the individual patient
2. check the scoliosis correction in all the three planes (frontal, sagittal and horizontal)
3. check the aesthetic correction
4. maximize brace tolerability (reduce visibility as much as possible for the used technique and allow movements and activity of daily life as much as possible)
5. apply all changes needed and, if necessary, even rebuild the brace without extra-charge for patients
6. check that the patient (and/or his/her parents) is able to fit the brace adequately
7. verify the patients mood and counsel him and the family at brace delivery

## New version

In each single check of a brace (case by case), the responsible physician with the orthotist has to:

1. verify accurately if it fits properly and fulfils the need of the individual patient
2. check the scoliosis correction in all the three planes (frontal, sagittal and horizontal)
3. check clinically the aesthetic correction
4. maximize brace tolerability (reduce visibility and allow movements and activity of daily life as as much as possible for the used technique)
5. apply all changes needed and, if necessary, even rebuild the brace without extra-charge for patients
6. check the corrections applied
7. check that the patient (and/or his/her parents) is able to apply or put on the brace properly
8. access the patient’s mood and counsel him and the family at brace delivery and at other follow-ups

## Votes

21. Do you agree with point #7 of recommendation #8 ?

- Yes
- No

22. Do you agree on all changes proposed to recommendation #8 ?

- Yes
- No

# Recommendation 9

## Old version

The check of each single brace has to be clinical and/or radiographic

## New version

The check by the treating physician of the efficacy of each single brace on each single patient has to be clinical, and radiographic if needed

## Votes

23. Do you agree on all changes proposed to recommendation #9 ?

- Yes
- No

# Recommendation 10

## Old version

The physician, orthotist and physical therapist has to check the brace each time they see the patient, as well as they have to check carefully patient compliance and reinforce the usefulness of brace treatment to the patient and his/her family

## New version

The physician, orthotist and physical therapist must check the brace and patient compliance regularly (MDs and CPOs each time they see the patient), and reinforce the usefulness of brace treatment to the patient and his/her family.

## Votes

24. Do you agree on all changes proposed to recommendation #10 ?

- Yes
- No

# Recommendation 11

## Old version

The physician has to follow-up the efficacy of the brace at least every 4 to 6 months, and eventually before if required by the orthotist and/or the physical therapist

## New version

The physician has to follow-up the braced patient regularly at least every 3 to 6 months. Standard intervals have to be reduced according to individual needs (first brace, growth spurt, progressive or atypical curve, poor compliance, request of other team members - CPO, PT …). Using tools (written protocols, recalls…) to make patients aware of their follow-up is strongly suggested

## Votes

25. Do you agree on all changes proposed to recommendation #11 ?

- Yes
- No

# Recommendation 12

## Old version

The brace has to be changed for a new one as soon as the child grows up, and this must be judged by the physician responsible for the treatment. Usually braces have to be changed every 12-24 months according to the growing phase, but even faster sometimes during the pubertal growth spurt

## New version

The brace has to be changed for a new one as soon as the child grows up or the brace loose efficacy, and this need can be suggested by the orthotist, but it is under the responsibility of the treating physician

## Votes

26. Do you agree on all changes proposed to recommendation #12 ?

- Yes
- No

# Recommendation 13

## Old version

The orthotist has to regularly check the fitting and comfort of the brace at least every 2-3 months, or even more frequently if required by the type of brace or by the patient. He has to check also for efficacy and, in front of any doubt, he has to contact the physician

## New version

The orthotist has to regularly check the brace. In front of any problem, he/she has to refer to the treating physician

## Votes

27. Do you agree with recommendation #13 ?

- Yes
- No

28. Do you think that recommendation #13 should be applied by clinicians engaged in future research in this field ?

- Yes
- No

29. Do you agree on all changes proposed to recommendation #13 ?

- Yes
- No

# Recommendation 14

## Old version

The physical therapist has to check the brace each time he/she sees the patient to propose the exercises needed for treatment. This means that she/he can identify problems of wearability or efficacy before the other members of the team and refer to the physician or orthotist respectively. If there are problems of compliance, or the need of more explanation to the patient or his/her family, he/she has to be trained to face all these problems and to give the correct answers. She/he is a member of the treating team, and he/she, as well as all professionals involved, has to behave consequently.

## New version

The physical therapist has to check the brace regularly. In front of any problem, she/he has to refer to the physician and not to the patient. As a member of the treating team, he/she has to be trained to face the problems of compliance, or the needs of more explanation by the patient or his/her family: in case she/he is not entirely a member of the treating team he must not act autonomously and must refer to the treating physician.

## Votes

30. Do you agree with recommendation #14 ?

- Yes
- No

31. Do you think that recommendation #14 should be applied by clinicians engaged in future research in this field ?

- Yes
- No

32. Do you agree on all changes proposed to recommendation #14 ?

- Yes
- No

# Your data (to be inserted in the paper, if you wish)

- Please indicate here, by a check mark, and omit your name below if you do not want your name inserted in the paper. Nevertheless, for statistical reasons, please **compile the anagraphical and professional information as outlined below.**

First and family name: _____________________________________________________________

Gender, age: _____________________________________________________________________

Profession: ______________________________________________________________________

Medical specialty (for MDs): ________________________________________________________

Number of braces prescribed and/or constructed and/or checked per year: _____________________

Number of year of experience in the field of scoliosis: ____________________________________
