# Supplementary material for: Guidelines on "Standards of management of idiopathic scoliosis with corrective braces in everyday clinics and in clinical research": SOSORT Consensus 2008
Source: Scoliosis. 2009 Jan 16;4:2. doi: 10.1186/1748-7161-4-2 (PMC2651850; doi:10.1186/1748-7161-4-2)
Supplement: Additional file 4 — Standards of management of idiopathic scoliosis with corrective braces in everyday clinics and in clinical research. The SOSORT Criteria for bracing. Standards of management of idiopathic scoliosis with corrective braces in everyday clinics and in clinical research. The SOSORT Criteria for bracing. [file 1748-7161-4-2-S4.doc]

# Standards of management of idiopathic scoliosis with corrective braces in everyday clinics and in clinical research

# The SOSORT Criteria for bracing

## Experience and competence

# Recommendation 1

The MD responsible for the treatment has to be experienced and should fulfil all these requirements:

1. training by a previous master (i.e. MD with at least 5 years of experience in bracing) for at least 2 years
2. at least 2 years of continuous practice in scoliosis bracing
3. prescription of at least 1 brace per working week (~45 per year) in the last 2 years
4. evaluation of at least 4 scoliosis patients per working week (~150 per year) in the last 2 years

Due to the actual situation of conservative treatment in many countries, this must be considered the ideal to be reached as soon as possible through education. Nevertheless, it must be recognised that experience and preparation is the only way to avoid problems to patients and reach adequate results in this field.

This recommendation has to be applied in everyday clinics and in research on clinical efficacy of bracing.

# Recommendation 2

The CPO constructing braces has to be experienced and should fulfil all these requirements

1. working continuously with a master MD (i.e. a MD fulfilling to recommendation 1 criteria) for at least 2 years
2. at least 2 years of continuous practice in scoliosis bracing
3. construction of at least 2 braces per working week (~100 per year) in the last 2 years

Due to the actual situation of conservative treatment in many countries, this must be considered the ideal to be reached as soon as possible through education. Nevertheless, it must be recognised that experience and preparation is the only way to avoid problems to patients and reach adequate results in this field

This recommendation has to be applied in everyday clinics and in research on clinical efficacy of bracing.

## Behaviours

# Recommendation 3

To ensure optimum results, the MD, CPO and physiotherapist (PT) must work together as a multiprofessional team. This can be accomplished, even if they are not currently located in the same workplace, through continuous exchange of information, team meetings, and verification of braces in front of single patients

This recommendation has to be applied in clinics and research.

# Recommendation 4

Commitment, time and counselling to increase compliance: MDs, CPOs and PTs have to give thorough advice and counselling to each single patient and family each time it is needed (at each contact for MDs and CPOs) provided they give as a team the same messages previously agreed

This recommendation has to be applied in everyday clinics and in research on clinical efficacy of bracing.

# Recommendation 5

All the phases of brace construction have to be followed for each single brace

1. prescription by a well trained and experienced MD (fulfilling recommendation 1 criteria)
2. construction by a well trained and experienced CPO (fulfilling recommendation 2 criteria)
3. check by the MD in team with the CPO, and possibly the PT
4. correction by the CPO according to MD indications
5. follow-up by the CPO, MD and PT

This recommendation has to be applied in everyday clinics and in research on clinical efficacy of bracing.

## Prescription

# Recommendation 6

In each single prescription of a brace (case by case), the MD must:

1. write the details of brace construction (where to push and where to leave space, how to act on the trunk to obtain results on the spine) when not already defined “a priori” with the CPO
2. prescribe the exact number of hours of brace wearing
3. be totally convinced of the brace proposed and committed to the treatment
4. use any ethical means to increase patient compliance, including thorough explanation of the treatment, aids such as photos, brochures, video, etc

This recommendation has to be applied in everyday clinics and in research on clinical efficacy of bracing.

## Construction

# Recommendation 7

In each single construction of a brace, case by case, the CPO has to:

1. check the prescription and its details and eventually discuss them with the prescribing MD, if needed, before construction
2. fully execute the agreed prescription
3. be totally convinced of the brace proposed and committed to the treatment
4. use any ethical means to increase patient compliance, including thorough explanation of the treatment, aids such as photos, brochures, video, etc

This recommendation has to be applied in clinics and research.

## Brace check

# Recommendation 8

In each single check of a brace,case by case, the responsible MD in partnership with the CPO has to:

1. verify accurately if it fits properly and fulfils the needs of the individual patient
2. check the scoliosis correction in all three planes (frontal, sagittal and horizontal)
3. check clinically the aesthetic correction
4. maximize brace tolerability (reduce visibility and allow movements and activity of daily life as much as possible for the chosen technique)
5. apply all changes needed and, if necessary, even rebuild the brace without extra-charge for patients
6. check the corrections applied
7. check that the patient (and/or his/her parents) is able to apply or put on the brace properly
8. access the patient’s mood and counsel him and the family at brace delivery and at other follow-ups

This recommendation has to be applied in clinics and research.

# Recommendation 9

The check of each single brace has to be clinical and/or radiographic

This recommendation has to be applied in clinics and research.

## Follow-up

# Recommendation 10

The MD, CPO and PT must check the brace and patient compliance regularly (MDs and CPOs each time they see the patient), and reinforce the usefulness of brace treatment to the patient and his/her family.

This recommendation has to be applied in clinics and research.

# Recommendation 11

The MD has to follow-up the braced patient regularly, at least every 3 to 6 months. Standard intervals have to be reduced according to individual needs (first brace, growth spurt, progressive or atypical curve, poor compliance, request of other team members - CPO, PT …). Using tools (written protocols, recalls…) to keep patients informed of their follow-up is strongly suggested.

This recommendation has to be applied in clinics and research.

# Recommendation 12

The brace has to be changed for a new one as soon as the child grows up or the brace loses efficacy, and this need can be suggested by the CPO, but is the responsibility of the treating MD

This recommendation has to be applied in clinics and research.

# Recommendation 13

The CPO has to regularly check the brace. In front of any problem, he/she has to refer to the treating MD

This recommendation has to be applied in everyday clinics and in research on clinical efficacy of bracing.

# Recommendation 14

The PT has to check the brace regularly. In response to any problem, she/he has to refer to the MD and not to the patient. As a member of the treating team, he/she has to be trained to face the problems of compliance, or the needs for more explanation by the patient or his/her family. In case she/he is not entirely a member of the treating team he must not act autonomously and must refer to the treating MD.

This recommendation has to be applied in clinics and research.
