# Supplementary material for: Guidelines on "Standards of management of idiopathic scoliosis with corrective braces in everyday clinics and in clinical research": SOSORT Consensus 2008
Source: Scoliosis. 2009 Jan 16;4:2. doi: 10.1186/1748-7161-4-2 (PMC2651850; doi:10.1186/1748-7161-4-2)
Supplement: Additional file 5 — Questionnaire to verify the achievement of the SOSORT Criteria for bracing: "Standards of management of idiopathic scoliosis with corrective braces in everyday clinics and in clinical research". Questionnaire to verify the achievement of the SOSORT Criteria for bracing: "Standards of management of idiopathic scoliosis with corrective braces in everyday clinics and in clinical research". This is now openly proposed to the general community both for clinical everyday use and research on clinical efficacy of bracing. [file 1748-7161-4-2-S5.doc]

# Questionnaire to verify the achievement of the SOSORT Criteria for bracing: “Standards of management of idiopathic scoliosis with corrective braces in everyday clinics and in clinical research”

This questionnaire has been developed to

- allow each professional to self-test if he satisfies recommended management criteria for bracing
- test in case of research studies if the management of patients has been adequate according to the actual standards
- help patients understand if their caregivers satisfy the actual management needs

Ideally all the answers to the questions should be “Yes”.

During the SOSORT Consensus, cumulative answers in terms of clinical behaviors were:

- 38% no negative answers
- 53% up to 1 negative answer
- 68% up to 5 negative answers
- 91% up to 8 negative answers

Consequently, provided all 44 answers are given (if it lacks one member of the team, all relative answers should be “no”), until new researches will refine the system, we propose

- Excellent: 0-1 no out of 44
- Good: 2-5 no out of 44
- Sufficient: 6-8 no out of 44
- Insufficient: 9 no or more out of 44

We are aware that these standards are not applicable everywhere in the world, currently, for many different reasons. Nevertheless, we strongly support their progressive application, and SOSORT is ready to support individuals and groups who need help in reaching these minimum standards through education and masterships.

For more information look at the journal Scoliosis ([www.scoliosisjournal.com](http://www.scoliosisjournal.com/)) where the original paper (Negrini S, Grivas TB, Kotwicki T, Rigo M, Zaina F. “Standards of management of idiopathic scoliosis with corrective braces in everyday clinics and in clinical research. SOSORT Consensus 2008”) has been published in 2008, and to the SOSORT web site ([www.sosort.org](http://www.sosort.org/)).

# All professionals as a team

1. Do you work in a multiprofessional team (physician, orthotist and eventually physiotherapist), through continuous exchange of information, team meetings, and verification of braces in front of single patients? No Yes
2. Do you give thorough advice and counselling to each single patient and family each time it is needed? No Yes
3. Do the different professionals in your team give the same, previously agreed messages to patients and families? No Yes
4. Do you check each single brace in team (physician, orthotist, and possibly physiotherapist)? No Yes
5. Do you follow-up regularly each single brace? No Yes
6. Do you access the patient’s mood and counsel him and the family at brace delivery and at other follow-ups? No Yes
7. Do you check each single brace clinically and/or radiographically? No Yes
8. Do you check the brace and patient compliance regularly and reinforce the usefulness of brace treatment to the patient and his/her family? No Yes

# Medical Doctors

1. Have you been trained by a previous master (i.e. a physician with at least 5 years of experience in bracing) for at least 2 years? No Yes
2. Did you have at least 2 years of continuous practice in scoliosis bracing? No Yes
3. Have you prescribed at least 1 brace per working week (~45 per year) in the last 2 years? No Yes
4. Have you evaluated at least 4 scoliosis patients per working week (~150 per year) in the last 2 years? No Yes
5. Do you prescribe each single brace to the constructing orthothist? No Yes
6. Do you write the details of brace construction (where to push and where to leave space, how to act on the trunk to obtain results on the spine) when not already defined “a priori” with the orthotist? No Yes
7. Do you prescribe the exact number of hours of brace wearing? No Yes
8. Are you totally convinced of the brace proposed and committed to the treatment? No Yes
9. Do you use any ethical mean to increase patient compliance, including thorough explanation of the treatment, aids such as photos, brochures, video, etc? No Yes
10. Do you verify accurately if the brace fits properly and fulfils the need of the individual patient? No Yes
11. Do you check the scoliosis correction in all the three planes (frontal, sagittal and horizontal)? No Yes
12. Do you check clinically the aesthetic correction? No Yes
13. Do you maximize brace tolerability (reduce visibility and allow movements and activity of daily life as much as possible for the used technique)? No Yes
14. Do you check the corrections applied? No Yes
15. Do you follow-up the braced patients regularly, at least every 3 to 6 months? No Yes
16. Do you reduce standard intervals according to individual needs (first brace, growth spurt, progressive or atypical curve, poor compliance, request of other team members)? No Yes
17. Do you take the responsibility to change the brace for a new one as soon as the child grows up or the brace loses efficacy? No Yes

# Orthotists

1. Have you been working continuously with a master physician (i.e. a physician fulfilling to recommendation 1 criteria) for at least 2 years? No Yes
2. Did you have at least 2 years of continuous practice in scoliosis bracing? No Yes
3. Have you constructed at least 2 braces per working week (~100 per year) in the last 2 years? No Yes
4. Do you construct each single brace according to physician prescription? No Yes
5. Do you correct each single brace according to physician indications? No Yes
6. Do you check the prescription and its details and eventually discuss them with the prescribing physician, if needed, before construction? No Yes
7. Do you fully execute the agreed prescription? No Yes
8. Are you totally convinced of the brace proposed and committed to the treatment? No Yes
9. Do you use any ethical mean to increase patient compliance, including thorough explanation of the treatment, aids such as photos, brochures, video, etc? No Yes
10. Do you maximize brace tolerability (reduce visibility and allow movements and activity of daily life as much as possible for the used technique)? No Yes
11. Do you apply all changes needed and, if necessary, even rebuild the brace without extra-charge for patients? No Yes
12. Do you suggest to change the brace for a new one as soon as the child grows up or the brace loses efficacy? No Yes
13. Do you check regularly the brace ? No Yes
14. In front of any problem with the brace, do you refer to the treating physician? No Yes

# Physiotherapists

1. Do you check the brace when you evaluate/treat a patient wearing a brace? No Yes
2. In front of any problem with the brace, do you refer to the treating physician? No Yes
3. In front of any problem with the brace, do you avoid to refer to the patient? No Yes
4. If you are a member of the treating team, have you been trained to face the problems of compliance, and the needs of explanation by the patient or his/her family? No Yes
5. If you are not a member of the treating team, do you avoid acting autonomously? No Yes
